# Comment on Kardos et al. Efficacy and Safety of a Single Ivy Extract Versus Two Herbal Extract Combinations in Patients with Acute Bronchitis: A Multi-Center, Randomized, Open-Label Clinical Trial. *Pharmaceuticals* 2025, *18*, 754

**DOI:** 10.3390/ph19040592

**Published:** 2026-04-08

**Authors:** Peter S. P. Cho

**Affiliations:** 1 Department of Respiratory Medicine, King’s College Hospital NHS Foundation Trust, London SE5 9RS, UK; p.cho@nhs.net; 2 Centre for Human and Applied Physiological Sciences, King’s College London, London SE1 1UL, UK

I read with interest the recently published open-label randomized trial comparing a single ivy extract with two herbal combination therapies (ivy/thyme and thyme/primrose) for the treatment of acute bronchitis [1]. Although bronchitis is a self-limiting illness, patients may benefit from herbal therapies, which have been shown to reduce cough severity and symptom burden [2]. In this trial, 325 adult patients were randomized 3:2:2 to receive ivy, ivy/thyme, or thyme/primrose for 7 days, followed by a 7-day observation period. The primary endpoint was the assessment of non-inferiority, and the secondary endpoint was the assessment of the superiority of ivy compared to each combination therapy, regarding the change from baseline in the Bronchitis Severity Score (BSS) at Day 7. The authors reported non-inferiority of the single ivy extract compared with both combination therapies and statistical superiority over ivy/thyme [1]. While the findings have value for hypothesis generation, I believe that the data are overinterpreted and require further validation, particularly given the emphasis on cross-study comparisons to support claims of comparative efficacy. The purpose of this letter is to provide a constructive appraisal of the study design, randomization strategy, and analytical approach, with a view to supporting a balanced understanding and informing future research.

My primary concern is that the open-label design, combined with a reliance on patient-reported outcomes (PRO) as endpoints, may have increased the potential for bias in symptom assessment. Blinding plays a key role in reducing such bias by helping to ensure that observed differences arise from treatment effects, rather than the expectations of participants or investigators [3]. In the absence of blinding, both patient perception and investigator evaluation may have been influenced by knowledge of the treatment received. The justification provided that a triple-dummy design would have been unduly burdensome appears limited, as blinding can be relatively straightforward. Indeed, blinding has been successfully implemented in previous acute bronchitis trials, and double- or triple-dummy designs are widely used in comparable respiratory studies, even in complex dosing scenarios [4,5,6,7].

The prospectively defined 3:2:2 randomization ratio resulted in unequal group sizes, with 140 participants assigned to the single ivy extract group, 93 to the ivy/thyme combination, and 95 to the thyme/primrose combination. While unequal allocation can occasionally be justified (e.g., to collect additional safety data), no rationale was provided for this approach, and such imbalance may affect both statistical power and the interpretability of results. Without explanation, the unequal allocation may imply that the sponsor’s product (ivy extract) was prioritized. The disproportionate allocation to the ivy extract arm may have revealed it as the experimental arm to participants and investigators. This may have inflated perceptions of experimental product superiority and introduced evaluation bias, particularly given the reliance on PROs as endpoints. In addition, the larger ivy group may have benefited from greater precision, potentially favoring the detection of apparent differences in efficacy compared with the smaller combination therapy comparator groups. Furthermore, treatment adherence was reportedly confirmed verbally, with no formal measure of product intake or compliance, leaving uncertainty regarding exposure comparability between treatment groups.

Validation and interpretation of clinical endpoints represent a key limitation of the study. The pre-defined non-inferiority margin of a BSS of 1.5 points, set at half the minimal clinically important difference (MCID), was used for the primary endpoint based on guidance from the German Institute for Quality and Efficiency in Healthcare [8]. To my knowledge, the BSS MCID has not been determined in acute bronchitis through robust (e.g., anchor- or distribution-based) methods, thus casting doubt on the clinical interpretability of the findings. By contrast, the Visual Analog Scale (VAS) and Leicester Cough Questionnaire (LCQ-acute) both have validated MCIDs in acute cough and may have been more valuable primary outcome measures [9]. Similarly, the Verbal Category Descriptive cough severity categories appear to lack formal validation, and their subjective wording may invite bias, as both patients and investigators could interpret the categories differently. The use of PROs, such as the BSS and VAS, is common and appropriate in studies of bronchitis. However, a more holistic assessment, such as a validated quality-of-life measure, could have been included to capture the broader impact of illness and recovery beyond cough-related endpoints. Such tools could also assess the value of the experimental phytotherapy as per the principles of value-based medicine [10].

Reporting of the analysis sets and key efficacy data was incomplete and, at times, inconsistent, reducing confidence in the overall findings. A greater alignment with CONSORT guidance, particularly regarding participant flow, would strengthen the study [11]. Of note, the authors did not report the reasons for trial discontinuation clearly. Key results (e.g., cough severity VAS and global efficacy) were presented with grouped p-values only, without corresponding effect sizes or confidence intervals, limiting interpretation of the magnitude and clinical relevance of the findings. Meanwhile, cough severity VAS data for the three phytotherapies were only presented in graphical format, thus limiting interpretation relative to the MCID (17 mm) in acute cough [9]. Taken together, the BSS and cough severity VAS outcomes were reported as significantly different between the three phytotherapies; however, considering the aforementioned bias, the clinical relevance of these findings may be limited. Other weaknesses include the inconsistent use of analysis sets for primary and secondary outcomes, and inaccuracies and omissions in the tables and figures.

Finally, in their discussion, the authors attempted to validate their findings using direct comparisons between their results and outcomes from independent randomized controlled trials [12,13,14]; however, these studies differ substantially from their own in terms of study designs, patient populations, and endpoints, making such comparisons inappropriate. By relying on these heterogeneous studies to support their conclusions, the authors risk overstating the strength of their evidence. Such cross-study comparisons should be interpreted with caution and cannot be used to substantiate claims of comparative efficacy.

In conclusion, while the recently published trial offers additional data on the use of phytotherapies for acute bronchitis, the methodological and reporting limitations undermine the robustness of the authors’ conclusions. The open-label design, unequal randomization, and incomplete reporting mean that the level of evidence presented is insufficient to substantiate claims of non-inferiority or superiority between treatments. While the findings may provide value for hypothesis generation, future larger-scale, double-blinded, randomized controlled trials are warranted to validate the findings and support meaningful conclusions regarding efficacy and relative treatment benefit. Such studies would ensure that the observed effects are substantiated without bias from the open-label design and unequal randomization.
